# Individual back-calculated size-at-age based on otoliths from Pacific coral reef fish species

**DOI:** 10.1038/s41597-020-00711-y

**Published:** 2020-10-27

**Authors:** Fabien Morat, Jérémy Wicquart, Nina M. D. Schiettekatte, Guillemette de Sinéty, Jean Bienvenu, Jordan M. Casey, Simon J. Brandl, Jason Vii, Jérémy Carlot, Samuel Degregori, Alexandre Mercière, Pauline Fey, René Galzin, Yves Letourneur, Pierre Sasal, Valeriano Parravicini

**Affiliations:** 1 PSL Université Paris: EPHE-UPVD-CNRS, USR 3278 CRIOBE, Université de Perpignan, 52 Avenue Paul Alduy, 66860 Perpignan, Cedex France; 2 Laboratoire d’Excellence “CORAIL”, EPHE, Perpignan, France; 3 Department of Biological Sciences, Simon Fraser University, Burnaby, BC V5A 1S6 Canada; 4 CESAB-FRB (Centre de synthèse et d’analyse sur la biodiversité), Institut Bouisson Bertrand, 5 rue de l’école de médecine, 34000 Montpellier, France; 5 Department of Ecology and Evolutionary Biology, University of California Los Angeles, Los Angeles, United States; 6 Université de la Nouvelle-Calédonie, Institut ISEA, BP R4, 98851 Nouméa Cedex, New Caledonia

**Keywords:** Ichthyology, Ageing, Population dynamics

## Abstract

Somatic growth is a critical biological trait for organismal, population, and ecosystem-level processes. Due to its direct link with energetic demands, growth also represents an important parameter to estimate energy and nutrient fluxes. For marine fishes, growth rate information is most frequently derived from sagittal otoliths, and most of the available data stems from studies on temperate species that are targeted by commercial fisheries. Although the analysis of otoliths is a powerful tool to estimate individual growth, the time-consuming nature of otolith processing is one barrier for collection of comprehensive datasets across multiple species. This is especially true for coral reef fishes, which are extremely diverse. Here, we provide back-calculated size-at-age estimates (including measures of uncertainty) based on sagittal otoliths from 710 individuals belonging to 45 coral reef fish species from French Polynesia. In addition, we provide Von Bertalanffy growth parameters which are useful to predict community level biomass production.

## Abstract (summary)

**Table:** 

Measurement(s)	growth
Technology Type(s)	otolithometry
Sample Characteristic - Organism	Actinopterygii
Sample Characteristic - Environment	coral reef
Sample Characteristic - Location	French Polynesia

Machine-accessible metadata file describing the reported data: 10.6084/m9.figshare.13027817

## Background & Summary

Anthropogenic disturbances, such as resource exploitation, pollution, and climate change, can significantly alter the structure and function of marine ecosystems^1–3^. Species differ in their contributions to ecological processes^4,5^; thus, accurately gauging the susceptibility of ecosystems to disturbances requires high-resolution data on life history traits across a broad suite of species, especially in highly diverse ecosystems^1,6,7^. Somatic growth, the increase of size (and weight) over time, is a critical trait to gauge biological processes that range from individuals to entire ecosystems. For fishes, this trait is particularly important because it links past, present, and future population trajectories in the context of fisheries and stock management; thus, it directly pertains to the provision of ecosystem services. Moreover, somatic growth rate is directly correlated with the energetic demands of organisms. As such, it underlies bioenergetic models that quantify energetic fluxes from individuals to ecosystems^8–10^, such as biomass production^11–13^ and nutrient cycling^14,15^. Quantifying somatic growth offers an opportunity to examine ecosystem function based on rates of ecological processes rather than employing traditional variables such as abundance or standing biomass^12,16^. Numerous temperate species have been extensively studied due to their commercial importance, but less information exists for the majority of coral reef species^17^. Reef fishes are extremely diverse, display a wide range of life history strategies, and provide an invaluable food source to millions of people in the world’s tropics. Therefore, a detailed understanding of reef fish growth rates is critical.

Fish growth parameters can be estimated using several approaches, but those that link age to body size are the most common. Growth can be measured from features preserved in hard structures, such as scales, vertebrae, fin spines, cleithra, opercula, and otoliths^18^. For teleost fishes, the most commonly used and reliable approach to estimate age is the analysis of growth rings found on otoliths. Otoliths are calcified structures of the inner ear that grow with the deposition of successive calcium carbonate layers, which respond to both circadian and seasonal rhythms^19–22^. Fish growth parameters can then be obtained with various models such as Gompertz, Logistic, or Von Bertalanffy (with the latter being the most commonly used approach)^23^. Such growth models can only be fitted based on a large number of individuals that cover the complete size range of the study species. However, due to the required sample sizes and the need for lethal sampling, obtaining such datasets is time consuming. Further, the raw data that permit size-at-age estimates are often unpublished, available only from technical reports, and/or available for a limited suite of commercial species. Multi-species growth curve comparisons are particularly rare, especially across a wide range of environmental conditions that may influence individual growth rates. Therefore, a back-calculation model that estimates fish size across previous ages based on otoliths represents an alternative to model growth^24^.

**Fig. 1:**
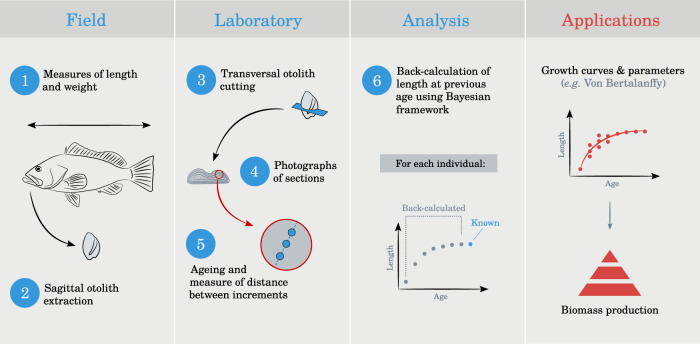
Illustration of the different steps that allowed the production of the dataset associated to this article.

Here, we provide a comprehensive dataset of raw otolith reads (51 species, 855 individuals) for corals reef fishes, collected across six islands in French Polynesia. Further, we provide the back-calculated size-at-age by species (45 species, 710 individuals); and by species across multiple locations (44 species, 669 individuals) using a Bayesian back-calculation model inspired by Vigliola and Meekan^24^. The inclusion of back-calculated size-at-age values alongside the raw data allows users to fit any regression model in line with their scientific question (Fig. 1). Finally, we provide Von Bertalanffy growth parameters estimated with Bayesian framework both by species and by species across multiple locations (when possible).

## Methods

### Study locations

**Fig. 2:**
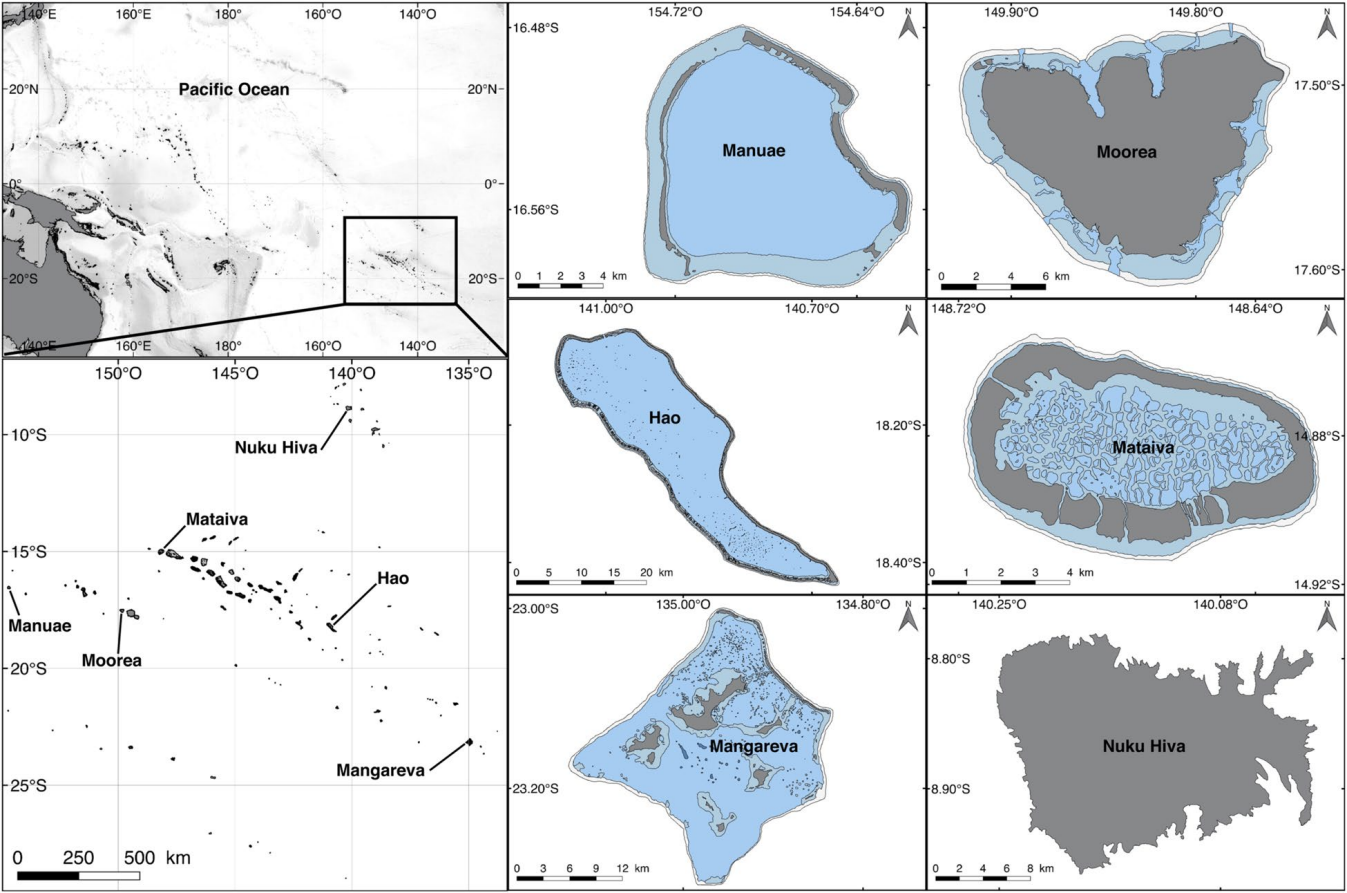
Map of sampling locations in French Polynesia.

Extending over 2,500,000 km^2^, French Polynesia includes 118 islands spread across five archipelagos: the Society Islands, Tuamotus, Marquesas, Austral Islands and Gambiers. We collected data across four archipelagos, including six distinct islands: Mo’orea and Manuae (Society Islands), Hao and Mataiva (Tuamotus), Mangareva (Gambiers), and Nuku Hiva (Marquesas) (Fig. 2). All fishes were collected in the lagoon and/or reef slope, depending on the accessibility of the respective habitats. Sea surface temperatures (SST) substantially varies around these six islands distributed across French Polynesia (Table 1).

**Table 1:** Minimum, mean, and maximum monthly average temperatures (°C) from 2002–2009, across the six locations where fishes were collected.

Island	Minimum	Mean	Maximum
Hao	25.72	27.53	29.26
Mangareva	23.20	25.44	27.74
Manuae	26.83	28.39	29.78
Mataiva	27.26	28.60	29.66
Moorea	26.62	28.29	30.94
Nuku Hiva	27.41	28.21	29.33

Temperatures are based on Bio-Oracle data^36^.

### Sampling design

Fishes were collected from Mo’orea (March 2016, March 2018, July 2018, and November 2018), Manuae (December 2014), and Nuku Hiva (August 2016 and March 2017) by spearfishing and clove oil, while fishes were collected from Hao (March 2017 and July 2017) and Mangareva (June 2018) only by spearfishing. Additional fishes from Mataiva were bought at the fish market in Tahiti. All applicable international, national, and/or institutional guidelines for the care and use of animals were followed.

#### Taxonomy and systematics

Fishes were identified using Bacchet *et al*.^25^ and Moore and Colas^26^.

#### Permits

Sample collection was permitted by the French Polynesian government (authorization number: 681MCE/ENV).

### Research methods

#### Field/Laboratory

In the laboratory, total length (TL) was measured to the nearest millimeter, and fishes were weighed to the nearest 0.1 grams. Then, pairs of sagittae (the largest otoliths of the inner ear) were extracted, cleaned with distilled water, dried, and stored in microtubes.

For each species, otoliths were cut transversely, using a diamond disc saw (Presi Mecatome T210) to obtain a section of 500 µm. Sections were then fixed on a glass side with thermoplastic glue (Crystalbond TM). Small otoliths were directly embedded in the thermoplastic glue and polished to obtain a transversal section. Otoliths were sanded with abrasive discs of decreasing grain size (2,400 and 1,200 grains cm^−2^) and polished with a 0.25 µm diamond suspension to reach the nucleus. All sections were photographed under a Leica DM750 light microscope with a Leica ICC50 HD microscope camera and LAS software (Leica Microsystems). When sections were too large for a single photograph, multiple photographs were taken and assembled with the software Photostitch (Canon).

A standardized transect across the otoliths (from the nucleus to the edge) was chosen for each species. On this transect, fish age was estimated and distances between annual growth increments were measured using the software ImageJ (Supplementary File 1). The age estimation was performed twice by two independent researchers to prevent biases induced by a single observer. When the coefficient of variation between the two observers was greater than 5%, a common reading was assessed for each section^21^.

#### Back-calculation

We then used a back-calculation procedure^24^ to estimate fish length at previous ages, which we modified to also quantify the uncertainty around the obtained length estimates. This method requires an examination of the shape of the relationship between the length at capture (*L*_*cpt*_) and the radius of the otolith at capture across all samples (*R*_*cpt*_) as follows:

1 $${L}_{cpt}={L}_{0p}-b{R}_{0p}^{c}+b{R}_{cpt}^{c}$$

where *L*_*0p*_ and *R*_*0p*_ are the fish size and radius of the otolith at hatching. The regression parameters *b* and *c* were estimated by fitting Bayesian models with RStan^27^. We used informative priors for both parameters [*b* ~ normal (200, 200) and *c* ~ normal (1, 1)].

For some individuals, it was not possible to measure the *R*_*0p*_ value. Nevertheless, these individuals were still included in the back-calculation model. To do so, we included all missing *R*_*0p*_ values as parameters in the model that are estimated in the posterior^28^. Specifically, these missing *R*_*0p*_ values were simultaneously modelled with the known *R*_*0p*_ values, so that their prior distribution was defined by the distribution of the known *R*_*0p*_ values. These prior distributions were then updated with the information provided by the aforementioned relationship (Eq. 1). Consequently, each missing *R*_*0p*_ value had a unique posterior distribution.

For all 4,000 iterations used to fit the models, we used parameters *b* and *c* (Eq. 1), to then quantify another parameter, the parameter *a*, combining both (Eq. 2).

2 $$a[i]={L}_{0p}-b\times {R}_{0p}{[i]}^{c}$$

Next, the back-calculation with the Modified Fry (MF) model (Eq. 3)^29^ was applied to quantify fish lengths at all ages for each individual, using parameter *a* for each iteration.

3 $${\rm{MF}}\,{\rm{model}}:{L}_{i}=a+exp\left(ln\left({L}_{0p}-a\right)+\frac{\left[ln\left({L}_{cpt}-a\right)-ln({L}_{0p}-a)\right]\left[ln\left({R}_{i}\right)-ln({R}_{0p})\right]}{\left[ln\left({R}_{cpt}\right)-ln({R}_{0p})\right]}\right)$$

where *L*_*i*_ and *R*_*i*_ are the fish length and otolith radius at age *i*, *L*_*0p*_ and *R*_*0p*_ are the fish size and radius of otolith at hatching. *L*_*0p*_ is provided for each species (Online-only Table 1).

We calculated *L*_*i*_ for the species that had sufficient replicates, and when possible also per species in each location separately. The estimation of parameters *b* and *c* (Eq. 1) required at least two values of *R*_*0p*_, so the back-calculation was not carried out when only one *R*_*0p*_ was available for a given species (or a given species in a certain location).

Individuals with estimated age at capture of one year where not used for back-calculation.

Finally, we reported the averages and standard deviations of those length estimates based on the 4,000 iterations. As such, the back-calculated estimates include a measure of uncertainty that can be integrated in the future applications.

#### Von bertalanffy growth curves

The Von Bertalanffy growth model (Eq. 4) is the most frequently used model to describe fish growth. This model is defined as:

4 $$Lt={L}_{\infty }\left(1-{e}^{-K\left(t-{t}_{0}\right)}\right)$$

where *Lt* is the average length at age *i*, *L*_*∞*_ is the asymptotic average length, *K* is the growth rate coefficient, and *t*_*0*_ is the age when the average length was zero. In order to validate the accuracy of our back-calculated size-at-age data, we compared growth curves fitted with raw data (total length at capture and estimated age at capture) to those fitted with back-calculated data. As back-calculated size-at-age data within individuals are highly auto-correlated, we designed a Bayesian hierarchical model that takes this auto-correlation into account by fitting individual growth curves as well as an average population-level growth curve. The model was applied on back-calculated data with at least five individuals and for individuals with an age at capture that was greater than two years.

We fitted models both for each species and for each species per location. In all models, we used informative priors for growth parameters extracted from FishBase (https://www.fishbase.se/search.php). We ran models with 2,000 iterations and a warmup of 1,000. When the $\widehat{R}$ was above one, indicating non-convergence of the Markov Chains Monte Carlo (MCMC), we ran models again augmenting iterations to 4,000 with a warmup of 2,000. If despite that, model convergence was still not achieved, we use MCMC chain plots of the model parameters to remove the individual(s) responsible for non-convergence.

As a comparison, we also ran a general non-linear Bayesian model on the raw data (*i.e*. using size and age at capture only). Back-calculated data contains more points (multiple points for each individual) than raw data (one point by individual), so the comparison was limited to the species with a sufficient number of individuals (n > 10) and age range in the raw data. These models were run using the package *brms*^30^.

All analyses were done with the software R v.3.6.3^31^ and the packages *rstan* (2.19.3), *tidyverse* (1.3.0)^32^, *plyr* (1.8.6)^33^, rfishbase (3.0.4)^34^, and *brms* (2.13.0)^30^.

## Data Records

The dataset is publicly accessible in the permanent figshare repository (10.6084/m9.figshare.12156159.v5)^35^. This dataset consists of:

1. 855 individuals from 51 fish species in 15 families collected across six locations in French Polynesia,
2. Fish total length and weight (when measured) for each individual,
3. Age estimations and back-calculated size-at-age for each individual, by species (45 species and 710 individuals) and by species across multiple locations (44 species, and 669 individuals).

## Technical Validation

**Fig. 3:**
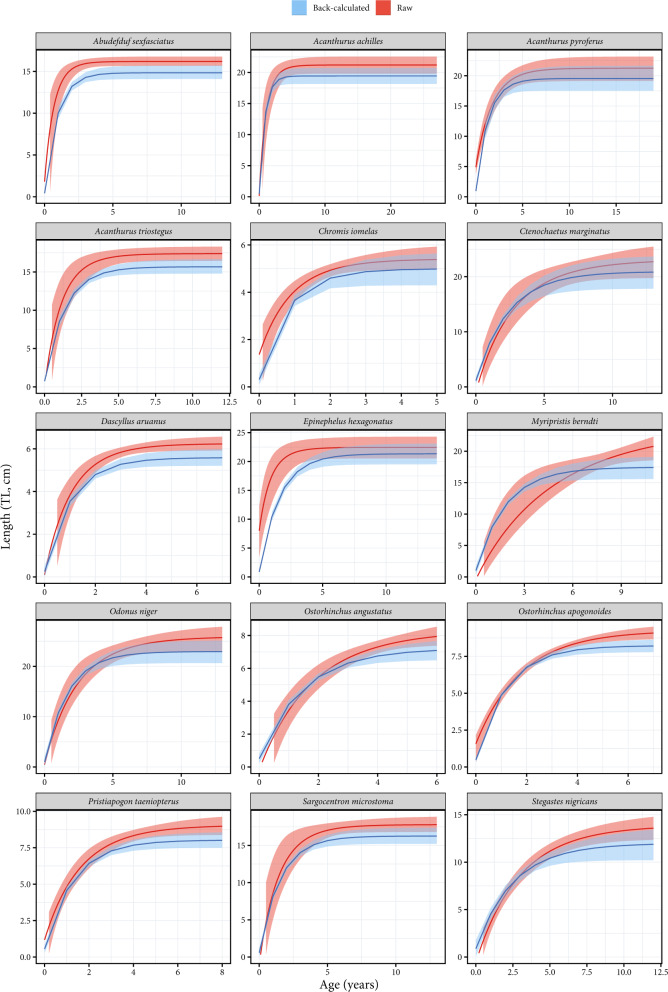
Comparison of Von Bertalanffy growth curves fitted on back-calculated size-at-age data (by species across multiple locations) to those fitted on raw data. The 15 species correspond to those with a sufficient number of individuals to fit the model on raw data.
- Further, Von Bertalanffy growth parameters estimated from otoliths were extracted from published articles, book chapters, reports and Ph.D. theses and compared to back-calculated parameters from our study (Online-only Table 3). For most species, the growth parameters from our study were similar to those in the literature. Differences may stem from different geographical locations (different temperatures, primary productivity, etc.), the number of analyzed fishes, different length measurements (standard, fork, or total length), or variations in modeling approaches.
- Finally, we compared our age estimates to the maximum ages reported in the literature (Online-only Table 3). Comparisons were possible for species with available data (seventeen species). Only five species were above the maximum reported age (*Caranx melanpygus, Cephalopholis urodeta, Chlorurus spilurus, Epinephelus merra, Plectropomus laevis*).

- The validity of fish names and families were verified on the World Register of Marine Species (WoRMS; http://www.marinespecies.org/index.php) and FishBase (https://www.fishbase.in/search.php).
- Each otolith was read twice by two readers to limit observer biases for age estimations. When the coefficient of variation between observers was greater than 5%, a common reading was assessed for each section^21^. Moreover, for each species, we provide a photograph of an otolith section with annual increments and reading axes (Supplementary File 1).
- To validate the accuracy of back-calculated data, growth curves fitted on back-calculated size-at-age were compared to those from raw data (total length at capture and estimated age at capture) (Fig. 3). This comparison was not possible for a species when the number of collected individuals was too low to fit a growth curve. Comparisons were possible for fifteen species, and for each of them, the 95% credible intervals overlapped between growth curves fitted on back-calculated data *versus* raw data, suggesting negligible differences between the two approaches (Fig. 3). Moreover, for all species, the curves from back-calculated data were always below those from raw data, indicating no overestimation of *L*_*∞*_. Further, because the back-calculated lengths also include the lengths at age zero, the length at hatching is more realistically represented in the regression models from the back-calculated data. Consequently, when using back-calculation, estimates of *K* tend to be higher and *L*_*∞*_ tend to be lower. The Von Bertalanffy growth parameters from our back-calculated size-at-age data by species and species across multiple locations are available online (Online-Only Table 2).

## Usage Notes

The dataset is provided as a csv file, which can be directly used by most statistical software. It contains eighteen variables, as described in Table 2. Additional growth parameters can be obtained by fitting other growth models (*e.g*. Gompertz model) using the variables ‘*Age*_*i*_’ and ‘*Li_sp_m*’ (species across all locations) or ‘*Li_sploc_m*’ (species by location).

**Table 2:** Description of the variables included in the dataset.

Column	Variable identity	Variable definition	Unit	Storage type	Range
1	*Family*	Family names according to fishbase (https://www.fishbase.de/search.php)	—	Factor	—
2	*Genus*	Genus names according to fishbase (https://www.fishbase.de/search.php)	—	Factor	—
3	*Species*	Species names according to fishbase (https://www.fishbase.de/search.php)	—	Factor	—
4	*ID*	Unique code identifying each individual	—	Factor	—
5	*Age*_*i*_	Age *i*	years	Integer	0–30
6	*R*_*i*_	Otolith radius at age *i*	mm	Numeric	0.008–3.784
7	*Age*_*cpt*_	Age at capture	years	Integer	0–30
8	*R*_*cpt*_	Otolith radius at capture	mm	Numeric	0.152–3.859
9	*L*_*cpt*_	Total length at capture	mm	Numeric	28.11–984.69
10	*L*_*0p*_	Total length at hatching	mm	Numeric	1.45–4.25
11	*R*_*0p*_	Otolith radius at hatching	mm	Numeric	0.008–0.136
12	*Li_sp_m*	Total length (mean) at age *i* calculated by species	mm	Numeric	1.45–949.65
13	*Li_sp_sd*	Standard deviation around the value of *Li_sp_m*	mm	Numeric	0–81.87
14	*Li_sploc_m*	Total length (mean) at age *i* calculated by species and location	mm	Numeric	1.45–948.67
15	*Li_sploc_sd*	Standard deviation around the value of *Li_sploc_m*	mm	Numeric	0–87.42
16	*Weight*	Wet body mass at capture	g	Numeric	0.4–12,950
17	*Location*	Island or archipelago of the sampling	—	Factor	—
18	*Observer*	Name of person that made the otolith reading	—	Factor	—

Back-calculated data are highly auto-correlated, so we recommend using a hierarchical structure to fit growth models.

Within the dataset, ‘NA’ indicates a missing value. Missing values are present for the variables ‘*R*_*i*_’ (n = 387), ‘*R*_*0p*_’ (n = 2,811), ‘*Li_sp_m*’ (n = 410), ‘*Li_sp_sd*’ (n = 410), ‘*Li_sploc_m*’ (n = 757), ‘*Lp_sploc_sd*’ (n = 757), and ‘*Weight*’ (n = 603). For the variable ‘*R*_*i*_’, missing values correspond to individuals for which it was impossible to estimate the radius at hatching from photographs. The ‘*R*_*0p*_’ values correspond to ‘*R*_*i*_’ values where ‘*Age*_*i*_’ is equal to zero. Because the ‘*R*_*0p*_’ value is the same for all ‘*Age*_*i*_’ of a given individual (‘ID’), a large number of NAs arises as soon as the ‘*R*_*i*_’ value is missing (where ‘*Age*_*i*_’ is equal to zero). For the variables ‘*Li_sp_m*’, ‘*Li_sp_sd*’, ‘*Li_sploc_m*’, and ‘*Lp_sploc_sd*’, missing values correspond to values with insufficient numbers of individuals or known ‘*R*_*0p*_’ measurements to accurately fit the Bayesian back-calculation model. The number of NAs for the variables ‘*Li_sp_m*’ and ‘*Li_sp_sd*’ (estimates by species) is lower than the number of NAs for the variables ‘*Li_sploc_m*’ and ‘*Lp_sploc_sd*’ (estimates for species by location). Finally, for the variable ‘*Weight’*, missing values are the result of missing sampling measurements.

## Supplementary information

Supplementary File 1

## Data Availability

The code to generate the back-calculated size-at-age data is available at https://github.com/JWicquart/fish_growth.

## Online-only Tables

**Online Only Table 1:** Fish size at hatching (*L*_*0p*_ in mm) for each species in the provided dataset.

Species	Family	*L*_*op*_	Level	Reference
*Abudefduf sexfasciatus* (Lacepède, 1801)	Pomacentridae	2.65	Species	^37^
*Acanthurus achilles* (Shaw, 1803)	Acanthuridae	1.70	Genus	^38^
*Acanthurus lineatus* (Linnaeus, 1758)	Acanthuridae	1.70	Genus	^38^
*Acanthurus nigricans* (Linnaeus, 1758)	Acanthuridae	1.70	Genus	^38^
*Acanthurus pyroferus* (Kittlitz, 1834)	Acanthuridae	1.70	Genus	^38^
*Acanthurus triostegus* (Linnaeus, 1758)	Acanthuridae	1.70	Species	^38^
*Balistapus undulatus* (Tilesius, 1820)	Balistidae	1.80	Family	^39^
*Caranx melampygus* (Cuvier, 1833)	Carangidae	3.15	Family	^39^
*Centropyge bispinosa* (Günther, 1860)	Pomacanthidae	1.95	Family	^39^
*Centropyge flavissima* (Cuvier, 1831)	Pomacanthidae	1.95	Family	^39^
*Cephalopholis argus* Schneider, 1801	Serranidae	1.90	Family	^39^
*Cephalopholis urodeta* (Forster, 1801)	Serranidae	1.90	Family	^39^
*Chaetodon citrinellus* (Cuvier, 1831)	Chaetodontidae	1.45	Genus	^39^
*Chaetodon ornatissimus* (Cuvier, 1831)	Chaetodontidae	1.45	Genus	^39^
*Cheilinus chlorourus* (Bloch, 1761)	Labridae	1.97	Genus	^40^
*Chlorurus spilurus* (Valenciennes, 1840)	Labridae (tribe Scarine)^41,42^	1.65	Family	^39^
*Chromis iomelas* (Jordan & Seale, 1906)	Pomacentridae	3.05	Family	^39^
*Chromis viridis* (Cuvier, 1830)	Pomacentridae	3.05	Family	^39^
*Ctenochaetus marginatus* (Valenciennes, 1835)	Acanthuridae	1.70	Family	^39^
*Ctenochaetus striatus* (Quoy & Gaimard, 1825)	Acanthuridae	1.70	Family	^39^
*Dascyllus aruanus* (Linnaeus, 1758)	Pomacentridae	2.10	Genus	^43^
*Dascyllus flavicaudus* (Randall & Allen, 1977)	Pomacentridae	2.10	Genus	^43^
*Epibulus insidiator* (Pallas, 1770)	Labridae	2.10	Family	^39^
*Epinephelus fasciatus* (Forsskål, 1775)	Serranidae	1.50	Species	^44^
*Epinephelus hexagonatus* (Forster, 1801)	Serranidae	1.70	Genus	^45–57^
*Epinephelus merra* (Bloch, 1793)	Serranidae	1.50	Species	^55^
*Epinephelus polyphekadion* (Bleeker, 1849)	Serranidae	1.65	Species	^51^
*Gnathodentex aureolineatus* (Lacepède, 1802)	Lethrinidae	1.55	Family	^39^
*Gymnosarda unicolor* (Rüppell, 1836)	Scombridae	2.75	Family	^39^
*Halichoeres trimaculatus* (Quoy & Gaimard, 1834)	Labridae	1.58	Genus	^58^
*Lutjanus fulvus* (Forster, 1801)	Lutjanidae	1.83	Genus	^59^
*Lutjanus gibbus* (Forsskål, 1775)	Lutjanidae	1.83	Genus	^59^
*Lutjanus kasmira* (Forsskål, 1775)	Lutjanidae	1.83	Species	^59^
*Monotaxis grandoculis* (Forsskål, 1775)	Lethrinidae	1.55	Family	^39^
*Mulloidichthys flavolineatus* (Lacepède, 1801)	Mullidae	2.50	Family	^39^
*Myripristis berndti* (Jordan & Evermann, 1903)	Holocentridae	1.80	Family	^39^
*Naso lituratus* (Forster, 1801)	Acanthuridae	1.70	Family	^39^
*Naso unicornis* (Forsskål, 1775)	Acanthuridae	1.70	Family	^39^
*Odonus niger* (Rüppell, 1836)	Balistidae	1.80	Family	^39^
*Ostorhinchus angustatus* (Smith & Radcliffe, 1911)	Apogonidae	4.25	Family	^39^
*Ostorhinchus apogonoides* (Bleeker, 1856)	Apogonidae	4.25	Family	^39^
*Parupeneus barberinus* (Lacepède, 1801)	Mullidae	1.95	Genus	^60^
*Plectropomus laevis* (Lacepède, 1801)	Serranidae	1.62	Genus	^61^
*Pristiapogon taeniopterus* (Bennett, 1836)	Apogonidae	4.25	Family	^39^
*Sargocentron microstoma* (Günther, 1860)	Holocentridae	1.80	Family	^39^
*Scarus psittacus* (Forsskål, 1775)	Labridae (tribe Scarine)	1.65	Family	^39^
*Siganus argenteus* (Quoy & Gaimard, 1825)	Siganidae	2.02	Genus	^62–65^
*Siganus spinus* (Linnaeus, 1758)	Siganidae	2.02	Genus	^62–65^
*Stegastes albifasciatus* (Schlegel & Müller, 1839)	Pomacentridae	3.05	Family	^39^
*Stegastes nigricans* (Lacepède, 1802)	Pomacentridae	3.05	Family	^39^
*Zebrasoma scopas* (Cuvier, 1829)	Acanthuridae	1.70	Family	^39^

The column *Level* refers to the taxonomic level at which *L*_*0p*_ was obtained. When not available, *L*_*0p*_ from different studies were averaged.

**Online-only Table 2:** Mean and 95% credible intervals of Von Bertalanffy growth parameters estimated with back-calculated data by species or by species across multiple locations.

Species	Location	n	*L*_*∞*_		*K*		*t*_*0*_	
			Mean	CI	Mean	CI	Mean	CI
*Abudefduf sexfasciatus*	All	10	14.851	14.131; 15.732	1.101	0.910; 1.326	−0.026	−0.073; 0.018
*Acanthurus achilles*	All	12	18.499	16.935; 20.205	1.185	0.924; 1.504	−0.023	−0.073; 0.023
*Acanthurus lineatus*	All	8	24.677	20.034; 29.844	1.044	0.666; 1.620	−0.029	−0.110; 0.034
*Acanthurus nigricans*	All	8	17.114	15.237; 19.098	1.485	1.111; 1.974	−0.011	−0.046; 0.019
*Acanthurus pyroferus*	All	6	19.594	17.620; 21.732	0.783	0.543; 1.082	−0.072	−0.225; 0.047
*Acanthurus triostegus*	All	17	15.717	15.025; 16.386	0.796	0.687; 0.920	−0.051	−0.110; 0.000
*Balistapus undulatus*	All	17	18.987	15.543; 22.894	0.338	0.244; 0.473	−0.282	−0.484; −0.108
	Marquesas	5	19.329	14.934; 23.825	0.413	0.213; 0.746	−0.254	−0.718; 0.004
	Moorea	12	17.235	13.713; 21.277	0.376	0.272; 0.527	−0.237	−0.447; −0.071
*Caranx melampygus*	All	7	54.386	46.496; 62.547	0.246	0.182; 0.335	−0.192	−0.376; −0.048
*Centropyge bispinosa*	All	6	5.240	3.328; 7.151	1.099	0.426; 2.329	−0.067	−0.144; −0.004
*Centropyge flavissima*	All	26	10.036	8.853; 11.393	0.400	0.288; 0.552	−0.222	−0.396; −0.085
	Gambiers	9	12.210	10.015; 15.044	0.204	0.138; 0.297	−0.834	−1.367; −0.394
	Marquesas	10	6.773	5.608; 7.889	1.076	0.769; 1.558	−0.033	−0.077; 0.005
	Moorea	5	8.175	6.766; 9.787	1.262	0.785; 1.955	−0.028	−0.085; 0.017
*Cephalopholis argus*	All	41	29.978	27.638; 32.385	0.397	0.357; 0.442	−0.251	−0.338; −0.174
	Gambiers	6	40.416	38.278; 42.529	0.297	0.241; 0.363	−0.450	−0.753; −0.206
	Hao	15	32.317	28.801; 35.914	0.401	0.340; 0.478	−0.184	−0.303; −0.078
	Moorea	10	22.852	18.391; 28.001	0.728	0.498; 1.066	−0.068	−0.186; 0.019
	Tuamotu	10	27.447	24.665; 30.458	0.439	0.328; 0.573	−0.225	−0.447; −0.056
*Cephalopholis urodeta*	All	8	19.922	16.821; 23.125	0.153	0.114; 0.208	−0.569	−0.800; −0.355
*Chaetodon ornatissimus*	All	9	14.554	13.403; 16.073	1.000	0.746; 1.311	−0.025	−0.103; 0.039
*Chlorurus spilurus*	All	26	23.283	21.416; 25.287	1.172	0.953; 1.446	−0.017	−0.057; 0.018
	Gambiers	13	22.585	20.510; 24.667	0.925	0.714; 1.188	−0.037	−0.104; 0.020
	Moorea	11	22.998	20.569; 25.664	1.245	0.895; 1.683	−0.016	−0.080; 0.037
*Chromis iomelas*	All	8	5.002	4.287; 5.759	1.292	0.885; 1.878	−0.053	−0.099; −0.016
*Chromis viridis*	All	6	12.002	9.944; 14.339	0.942	0.596; 1.431	−0.050	−0.164; 0.036
*Ctenochaetus marginatus*	All	14	20.925	18.051; 23.972	0.424	0.348; 0.512	−0.134	−0.223; −0.049
*Ctenochaetus striatus*	All	26	17.145	16.388; 17.985	0.849	0.75; 0.958	−0.045	−0.091; −0.006
	Gambiers	11	16.990	15.935; 18.097	0.566	0.475; 0.674	−0.112	−0.215; −0.020
	Moorea	15	17.326	16.203; 18.444	1.142	0.966; 1.344	−0.021	−0.055; 0.010
*Dascyllus aruanus*	All	16	5.596	5.234; 6.008	0.963	0.781; 1.175	−0.050	−0.102; −0.005
*Dascyllus flavicaudus*	All	8	8.977	8.145; 9.873	0.499	0.400; 0.615	−0.153	−0.272; −0.047
*Epibulus insidiator*	All	17	22.532	20.262; 25.000	0.565	0.458; 0.698	−0.113	−0.198; −0.038
*Epinephelus fasciatus*	All	10	20.424	17.269; 23.613	0.715	0.541; 0.949	−0.038	−0.099; 0.017
*Epinephelus hexagonatus*	All	13	21.301	19.486; 23.193	0.637	0.516; 0.781	−0.067	−0.158; 0.010
*Epinephelus merra*	All	44	17.755	16.282; 19.260	0.854	0.752; 0.972	−0.045	−0.083; −0.010
	Gambiers	13	18.520	16.848; 20.277	0.655	0.514; 0.824	−0.093	−0.206; 0.001
	Hao	11	22.481	21.199; 23.666	0.842	0.696; 1.021	−0.043	−0.115; 0.018
	Moorea	20	14.078	12.445; 15.856	1.192	1.027; 1.413	−0.016	−0.045; 0.010
*Epinephelus polyphekadion*	All	14	38.922	34.259; 44.032	0.281	0.223; 0.355	−0.236	−0.384; −0.101
*Gnathodentex aureolineatus*	All	8	20.184	18.372; 22.017	0.549	0.412; 0.716	−0.154	−0.319; −0.024
*Halichoeres trimaculatus*	All	5	17.999	15.054; 22.165	0.645	0.396; 0.952	−0.036	−0.162; 0.064
*Lutjanus fulvus*	All	12	23.880	21.968; 25.709	0.674	0.532; 0.859	−0.086	−0.189; 0.000
	Moorea	6	21.820	18.331; 24.992	1.175	0.809; 1.764	−0.019	−0.073; 0.026
	Tuamotu	6	24.765	22.022; 27.703	0.476	0.347; 0.638	−0.215	−0.468; −0.030
*Mulloidichthys flavolineatus*	All	14	25.756	24.711; 26.801	1.601	1.402; 1.828	−0.008	−0.027; 0.009
*Myripristis berndti*	All	30	20.121	18.629; 21.580	0.419	0.359; 0.489	−0.235	−0.342; −0.139
	Gambiers	7	23.324	20.284; 26.325	0.338	0.244; 0.463	−0.391	−0.748; −0.130
	Marquesas	8	21.795	20.828; 22.762	0.378	0.313; 0.456	−0.298	−0.513; −0.116
	Moorea	15	17.458	15.582; 19.219	0.553	0.442; 0.696	−0.110	−0.201; −0.030
*Naso lituratus*	All	14	31.222	27.811; 34.849	1.815	1.409; 2.339	−0.004	−0.023; 0.013
*Odonus niger*	All	15	25.063	22.646; 27.522	0.460	0.369; 0.576	−0.141	−0.247; −0.051
*Ostorhinchus angustatus*	All	13	7.229	6.567; 7.951	0.681	0.529; 0.851	–0.111	−0.188; −0.047
*Ostorhinchus apogonoides*	All	20	8.243	7.819; 8.703	0.836	0.735; 0.947	−0.072	−0.100; −0.045
*Plectropomus laevis*	All	25	71.926	65.904; 77.758	0.317	0.265; 0.380	−0.194	−0.311; −0.091
	Gambiers	12	77.388	71.296; 83.567	0.253	0.211; 0.302	−0.408	−0.609; −0.229
	Hao	13	52.309	47.305; 57.358	0.682	0.583; 0.795	−0.012	−0.049; 0.024
*Pristiapogon taeniopterus*	All	25	8.013	7.488; 8.580	0.780	0.656; 0.922	−0.093	−0.151; −0.043
*Sargocentron microstoma*	All	18	15.714	14.113; 17.340	0.701	0.567; 0.864	−0.049	−0.100; −0.002
*Siganus argenteus*	All	11	29.483	24.864; 35.111	0.728	0.491; 1.053	−0.036	−0.130; 0.044
*Stegastes nigricans*	All	9	11.721	10.733; 12.95	0.452	0.349; 0.573	−0.160	−0.321; −0.023

CI = lower and upper 95% credible interval bounds. All = all locations; n = number of individuals. *L*_*∞*_ is in cm (total length), *K* in year^−1^, and *t*_*0*_ is the theoretical age when the average length was zero.

**Online-only Table 3:** Comparison of Von Bertalanffy growth parameters (*L*_*∞*_ *K* and *t*_*0*_) maximum age (in years) and size between results of this study (by species) and those available in the literature.

Species	*L*_*∞*_	*K*	*t*_*0*_	Maximum age	Maximum size	Reference
*Abudefduf sexfasciatus*	14.851	1.101	−0.026	13	17.2	This study
*Acanthurus achilles*	18.499	1.185	−0.023	27	24.6	This study
*Acanthurus lineatus*	24.677	1.044	−0.029	23	36.2	This study
	18.274	0.462	−0.32		20.6	^66^
	22.1	0.12	−15.6	12	22.9	^67^
	18.909	0.68		42		^68^
	17.484	0.34		25		^68^
	17	0.416	−0.329		20	^69^
*Acanthurus nigricans*	17.114	1.485	−0.011	9	21.0	This study
	14.286	0.28	−0.69		16	^66^
*Acanthurus pyroferus*	19.594	0.783	−0.072	19	23.0	This study
*Acanthurus triostegus*	15.717	0.796	−0.051	12	19.4	This study
*Balistapus undulatus*	18.987	0.338	−0.282	18	28.3	This study
*Caranx melampygus*	54.386	0.246	−0.192	15	71.3	This study
	89.7	0.233	−0.044	6	66	^70^
*Centropyge bispinosa*	5.24	1.099	−0.067	11	8.0	This study
*Centropyge flavissima*	10.036	0.4	−0.222	27	15.0	This study
*Cephalopholis argus*	29.978	0.397	−0.251	21	45.0	This study
	50.6	0.075	−6.5	25	54.2	^71^
	48.5499	0.049	−14.145	39	48.5	^72^
	44.22	0.26	1.33	6	38.7	^73^
	39.901	0.271	−0.169	24		^74^
	37.119	0.339	−0.147	14		^74^
	31.44	0.255	0	15	32.2	^75^
*Cephalopholis urodeta*	19.922	0.153	−0.569	17	21.4	This study
	20.645	0.31		12		^76^
	14.5	1.39		10		^77^
*Chaetodon ortissimus*	14.554	1	−0.025	10	17.5	This study
*Chlorurus spilurus*	23.283	1.172	−0.017	16	34.4	This study
	34.4	0.4	−0.13	11		^78^
	21.8	0.95	−0.075	9	26.5	^79^
*Chromis iomelas*	5.002	1.292	−0.053	5	6.1	This study
*Chromis viridis*	12.002	0.942	−0.05	9	14.8	This study
*Ctenochaetus margitus*	20.925	0.424	−0.134	13	27.0	This study
*Ctenochaetus striatus*	17.145	0.849	−0.045	17	21.8	This study
	17.2	0.75	−0.4	34	21.4	^80^
	18.2	0.5	−0.6	26	22	^80^
	17.6	1.4	−0.2	36	21.4	^80^
	17.8	0.7	−0.4	21	20.5	^80^
	15.9	0.5	−0.7	17	17.8	^80^
	12.8	0.6	−0.7	28	15.8	^80^
	19.4	1	−0.3	37	23	^80^
	17.1	0.9	−0.4	29	20.2	^80^
	20.1	0.8	−0.3	32	22.8	^80^
	19.9	0.3	−0.8	34	23.3	^80^
	23.2	1.3	−0.2	28	25.9	^80^
	20.5	2.1	−0.1	19	23.8	^80^
	18.5	1.1	−0.3	21	20.9	^80^
	18.8	0.4	−0.6	27	25.2	^80^
	19.4	0.4	−0.7	20	21	^80^
	16.8	0.591	−0.27		19.7	^66^
	13.2	0.66	−0.16			^81^
	11.9	0.72	−0.12			^81^
	12.5	0.49	−0.36			^81^
	11.9	0.72	−0.12			^81^
	12.6	0.69	−0.05			^81^
	11.9	0.72	−0.12			^81^
	17	0.9	0.2	34	21	^82^
	25.6	0.424	−0.643		21	^69^
	16.92	0.63	0	19	20.2	^83^
	15.75	1.09	0	17	19.5	^84^
	16.8	0.98	0	6	18.8	^85^
	15.05	1.318	0	11	17.1	^75^
*Dascyllus aruanus*	5.596	0.963	−0.05	7	7.1	This study
*Dascyllus flavicaudus*	8.977	0.499	−0.153	13	10.4	This study
*Epibulus insidiator*	22.532	0.565	−0.113	16	35.0	This study
	19.894	0.25	−0.172	16	22.5	^86^
*Epinephelus fasciatus*	20.424	0.715	−0.038	13	25.6	This study
	28.1814	0.405	−0.685	21	44	^72^
	28.213	0.469	−0.198	16		^74^
	27.46	0.45	−0.212	12		^74^
*Epinephelus hexagotus*	21.301	0.637	−0.067	14	26.8	This study
*Epinephelus merra*	17.755	0.854	−0.045	17	26.1	This study
	21.2	0.5	−0.009	6	28	^87^
	16.46	1.01	0	6	20.8	^83^
*Epinephelus polyphekadion*	38.922	0.281	−0.236	20	51.4	This study
	44.71	0.251	−0.14	22	54	^88^
	57.9	0.18	0		62	^89^
	56.58	0.139	−1.18	26	61.8	^90^
	56.2345	0.194	−0.081	44	69.1	^72^
	53.701	0.195	−0.245	33		^74^
	58.237	0.206	−0.212	24		^74^
*Gthodentex aureolineatus*	20.184	0.549	−0.154	17	25.5	This study
*Halichoeres trimaculatus*	17.999	0.645	−0.036	5	18.6	This study
*Lutjanus fulvus*	23.88	0.674	−0.086	21	28.9	This study
	26.5	0.41	−0.49	34	33.2	^91^
	20.34	1.306	0	19	28.7	^75^
	23.62	0.735	0	19	28.7	^75^
*Mulloidichthys flavolineatus*	25.756	1.601	−0.008	6	31.1	This study
	38	0.27	−1.15	6	33.4	^92^
*Myripristis berndti*	20.121	0.419	−0.235	24	27.9	This study
*Naso lituratus*	31.222	1.815	−0.004	10	43.2	This study
	21.2	1.38	−0.2	14	26.3	^17^
	20.4	0.93	−0.3	13	25.1	^17^
	27.05	0.92	0	20	30.1	^83^
	23.22	1.05	0	20	30.1	^83^
	26.59	0.79	0	20	28.1	^84^
	23.43	1.11	0	20	28.1	^84^
	23.2	1.1	0	17	23.6	^85^
	19.9	1.92	0	17	23.6	^85^
	23.77	1.171	0	13	25	^75^
	20.72	2.055	0	13	25	^75^
*Odonus niger*	25.063	0.46	−0.141	16	35.0	This study
*Ostorhinchus angustatus*	7.229	0.681	−0.111	6	8.3	This study
*Ostorhinchus apogonoides*	8.243	0.836	−0.072	7	9.6	This study
*Plectropomus laevis*	71.926	0.317	−0.194	22	95.7	This study
	101.5	0.19	0		108.1	^89^
	115.9	0.096	−2.28	16	115	^93^
*Pristiapogon taeniopterus*	8.013	0.78	−0.093	8	10.4	This study
*Sargocentron microstoma*	15.714	0.701	−0.049	13	20.4	This study
*Siganus argenteus*	29.483	0.728	−0.036	13	38.8	This study
	27.4	0.9	−0.3		33.3	^94^
*Stegastes nigricans*	11.721	0.452	−0.16	13	13.8	This study

*L*_*∞*_ and maximum size in this study are expressed as total length (cm). Fish lengths from the literature are in cm and can be either expressed in total, standard, or fork length. *K* is in year^−1^ and *t*_*0*_ is the theoretical age when the average length was zero.
