# Supplementary material for: Individual back-calculated size-at-age based on otoliths from Pacific coral reef fish species
Source: Sci Data. 2020 Oct 27;7:370. doi: 10.1038/s41597-020-00711-y (PMC7591892; doi:10.1038/s41597-020-00711-y)
Supplement: Supplementary file 1 [file 41597_2020_711_MOESM1_ESM.pdf]

Supplementary File 1

Examples of transverse sagittal otolith sections with the increment patterns (denoted by white marks) used for the back-calculation of size-at-age for the species studied. The numbers correspond to [ACANTHURIDAE] 1. *Acanthurus achilles* (16 years, 192.84 mm TL), 2. *Acanthurus lineatus* (8 years, 259 mm TL), 3. *Acanthurus nigricans* (5 years, 150 mm TL), 4. *Acanthurus pyroferus* (7 years, 225 mm TL), 5. *Acanthurus triostegus* (8 years, 181.72 mm TL), 6. *Ctenochaetus marginatus* (8 years, 233.36 mm TL), 7. *Ctenochaetus striatus* (13 years, 175 mm TL), 8. *Naso lituratus* (10 years, 300 mm TL), 9. *Naso unicornis* (17 years, 612.65 mm TL), 10. *Zebrasoma scopas* (12 years, 142 mm TL), [APOGONIDAE] 11. *Ostorhinchus angustatus* (6 years, 83.009 mm TL), 12. *Ostorhinchus apogonoides* (4 years, 89.2 mm TL), 13. *Pristiapogon taeniopterus* (3 years, 70.012 mm TL), [BALISTIDAE] 14. *Balistapus undulatus* (17 years, 203.955 mm TL), 15. *Odonus niger* (3 years, 181.96 mm TL), [CARANGIDAE] 16. *Caranx melampygus* (15 years, 712.497 mm TL), [CHAETODONTIDAE] 17. *Chaetodon citrinellus* (2 years, 65.887 mm TL), 18. *Chaetodon ornatissimus* (7 years, 139 mm TL), [HOLOCENTRIDAE] 19. *Myripristis berndti* (11 years, 255 mm TL), 20. *Sargocentron microstoma* (3 years, 64.271 mm TL), [LABRIDAE] 21. *Cheilinus chlorourus* (6 years, 205.35 mm TL), 22. *Epibulus insidiator* (12 years, 284 mm TL), 23. *Halichoeres trimaculatus* (5 years, 171 mm TL), [LETHRINIDAE] 24. *Gnathodentex aureolineatus* (13 years, 224.16 mm TL), 25. *Monotaxis grandoculis* (20 years, 427.44 mm TL), [LUTJANIDAE] 26. *Lutjanus fulvus* (12 years, 284.014 mm TL), 27. *Lutjanus gibbus* (1 years, 135 mm TL), 28. *Lutjanus kasmira* (30 years, 326.26 mm TL), [MULLIDAE] 29. *Mulloidichthys flavolineatus* (4 years, 257.481 mm TL), 30. *Parupeneus barberinus* (6 years, 486.78 mm TL), [POMACANTHIDAE] 31. *Centropyge bispinosa* (7 years, 62.105 mm TL), 32. *Centropyge flavissima* (8 years, 109.44 mm TL), 33. *Abudefduf sexfasciatus* (7 years, 159 mm TL), 34. *Chromis iomelas* (2 years, 40.831 mm TL), 35. *Chromis viridis* (9 years, 135 mm TL), 36. *Dascyllus aruanus* (6 years, 56.828 mm TL), 37. *Dascyllus flavicaudus* (13 years, 102 mm TL), 38. *Stegastes albifasciatus* (1 years, 58.533 mm TL), 39. *Stegastes nigricans* (13 years, 125 mm TL), [LABRIDAE, previously SCARIDAE] 40. *Chlorurus spilurus* (16 years, 295 mm TL), 41. *Scarus psittacus* (3 years, 279.836 mm TL), [SCOMBRIDAE] 42. *Gymnosarda unicolor* (7 years, 984.69 mm TL), [SERRANIDAE] 43. *Cephalopholis argus* (13 years, 430 mm TL), 44. *Cephalopholis urodeta* (14 years, 150 mm TL), 45. *Epinephelus fasciatus* (9 years, 250 mm TL), 46. *Epinephelus hexagonatus* (7 years, 220 mm TL), 47. *Epinephelus merra* (16 years, 200 mm TL), 48. *Epinephelus polyphekadion* (9 years, 366 mm TL), 49. *Plectropomus laevis* (10 years, 654.82 mm TL), [SIGANIDAE] 50. *Siganus argenteus* (7 years, 327.12 mm TL), 51. *Siganus spinus* (2 years, 220.861 mm TL).

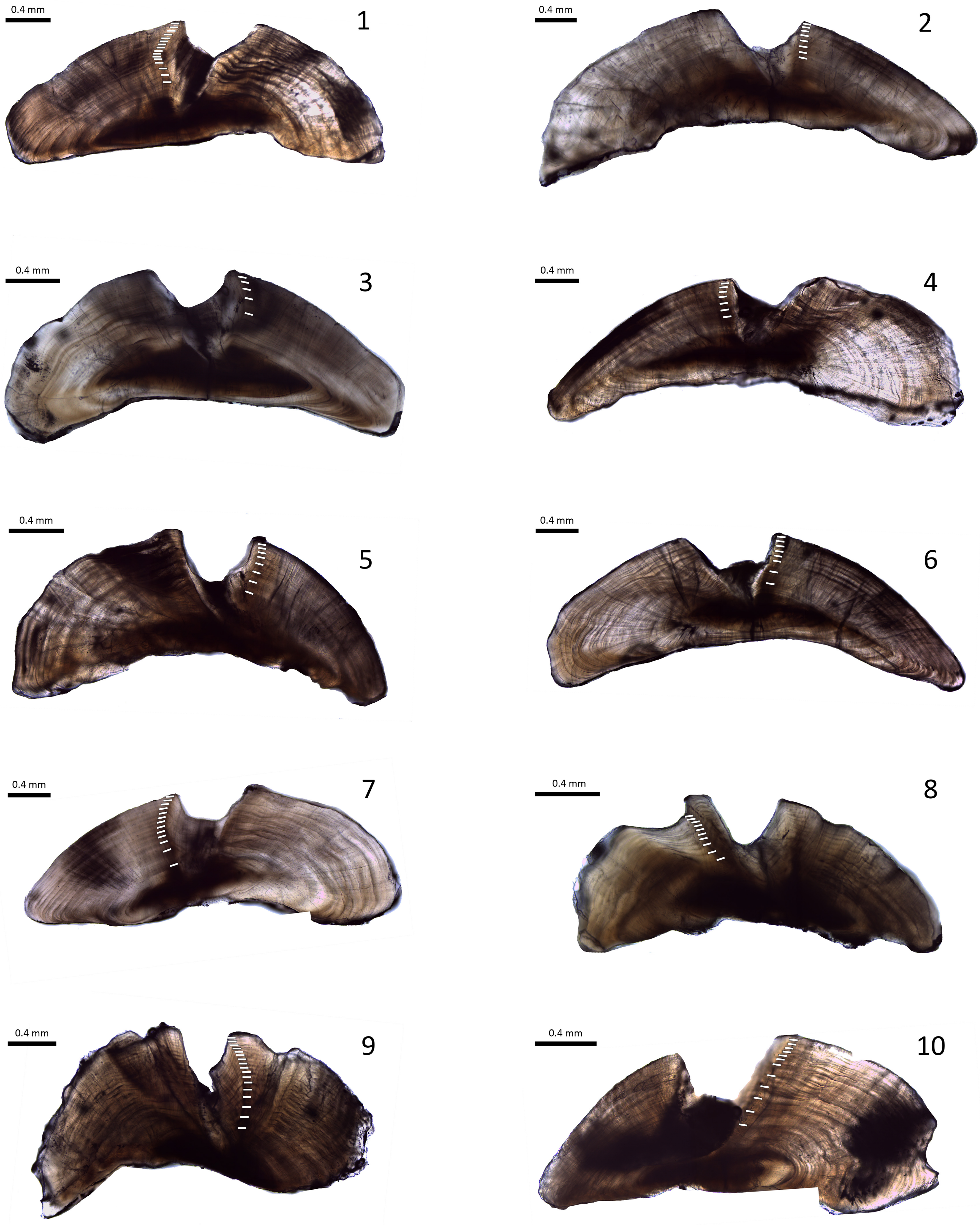
0.4 mm
1
0.4 mm
2
0.4 mm
3
0.4 mm
4
0.4 mm
5
0.4 mm
6
0.4 mm
7
0.4 mm
8
0.4 mm
9
0.4 mm
10

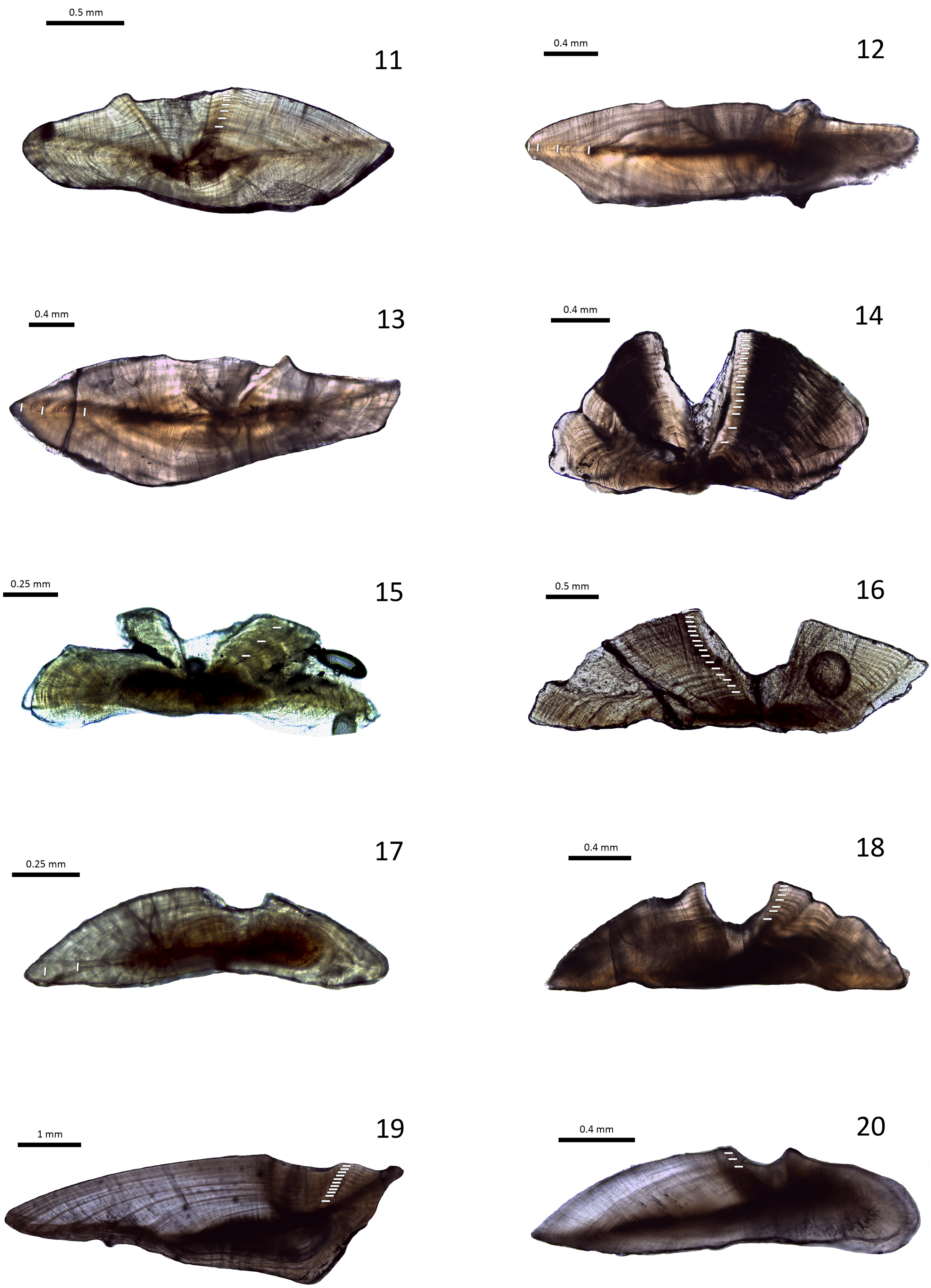
0.5 mm
11
0.4 mm
12
0.4 mm
13
0.4 mm
14
0.25 mm
15
0.5 mm
16
0.25 mm
17
0.4 mm
18
1 mm
19
0.4 mm
20

21

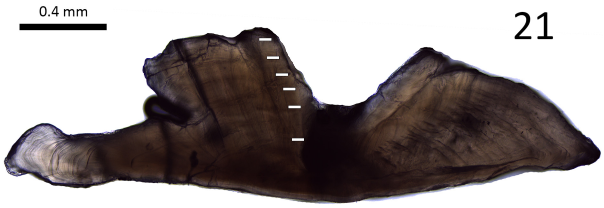

22

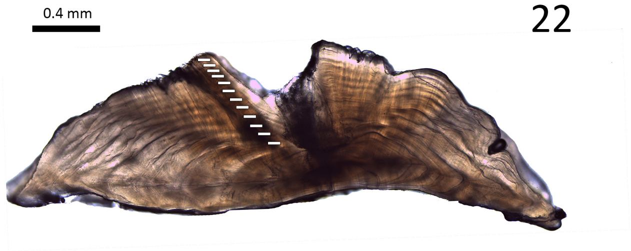

23

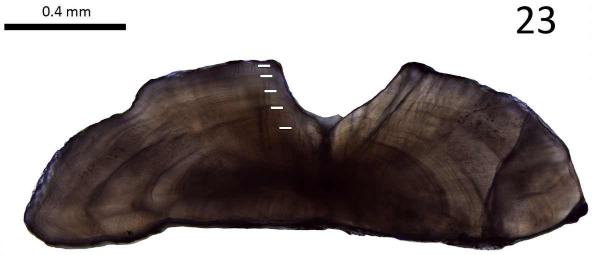

24

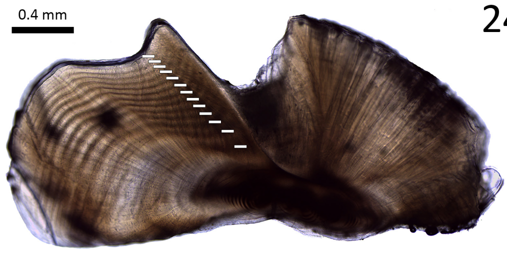

25

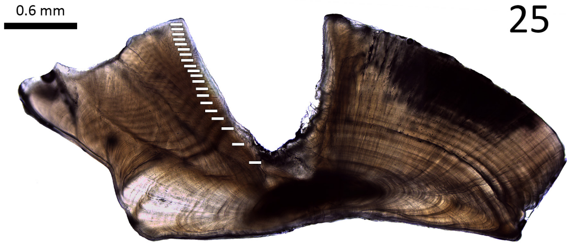

26

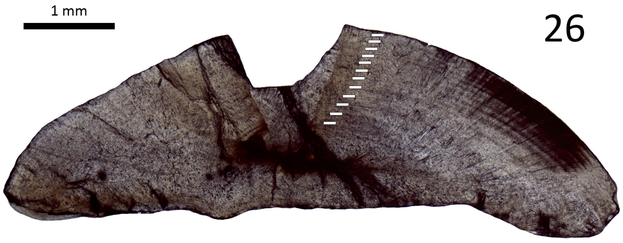

27

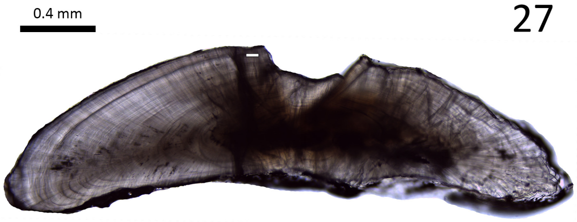

28

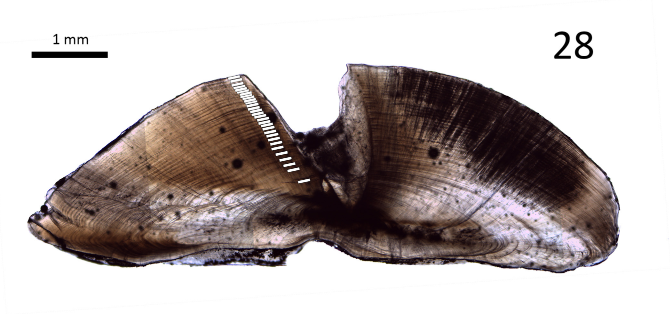

29

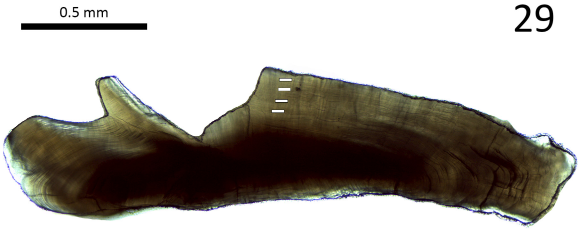

30

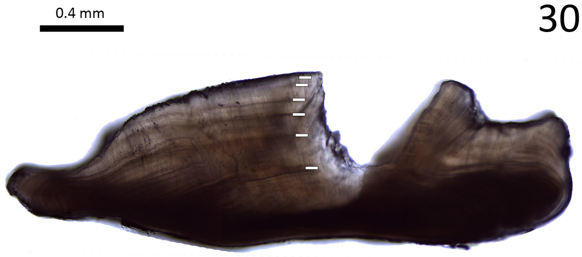

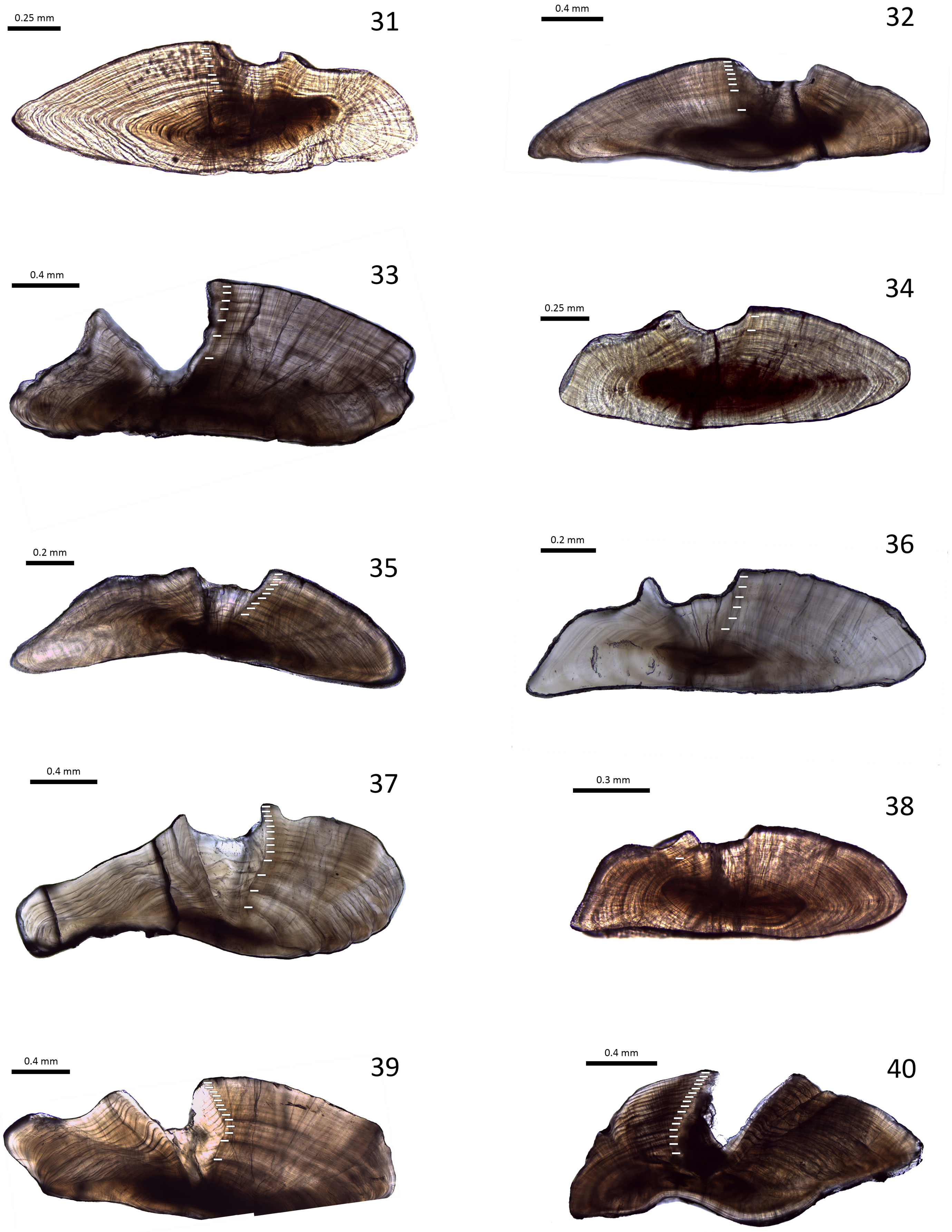
0.25 mm
31
0.4 mm
32
0.4 mm
33
0.25 mm
34
0.2 mm
35
0.2 mm
36
0.4 mm
37
0.3 mm
38
0.4 mm
39
0.4 mm
40

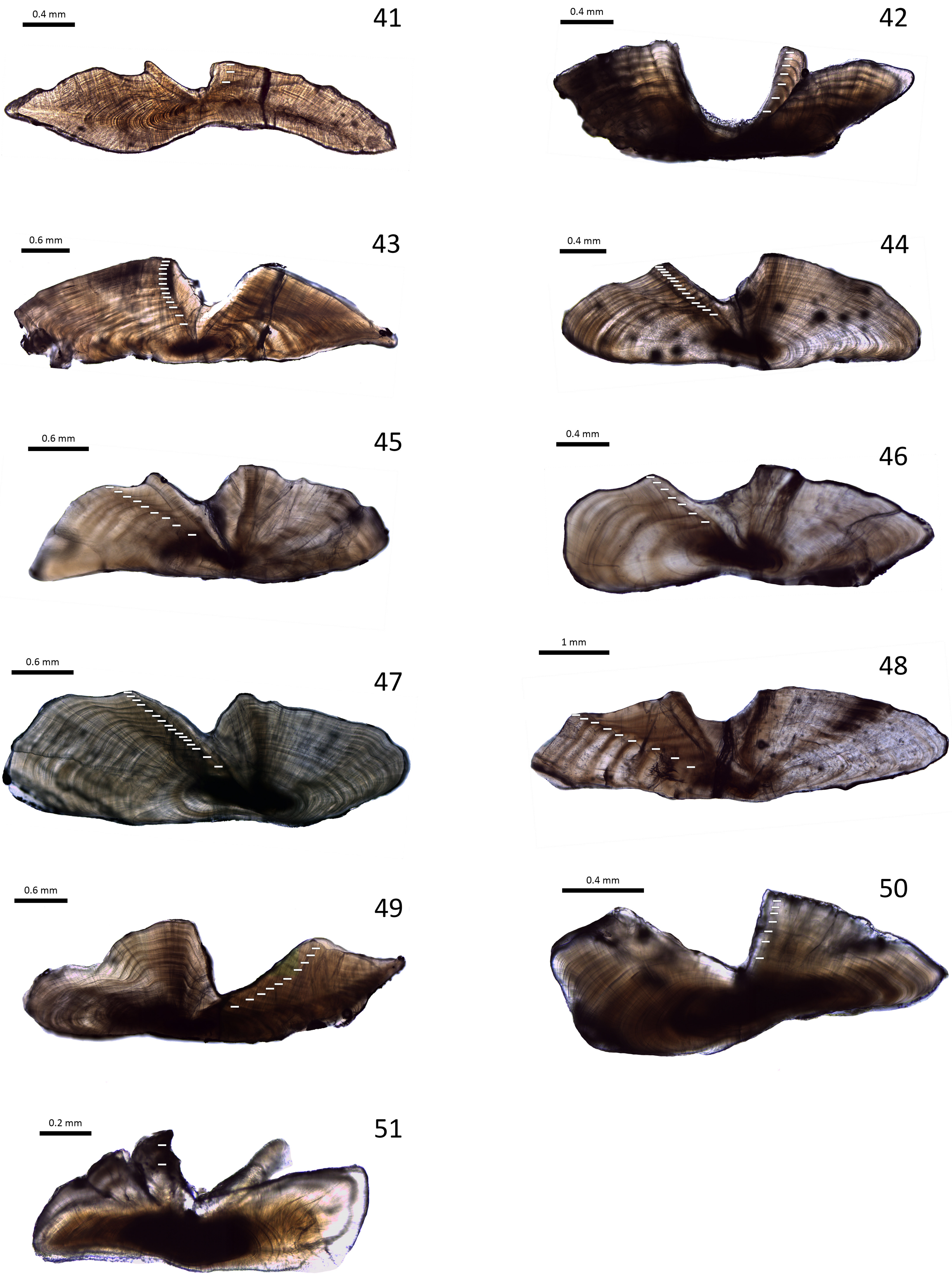
0.4 mm
41
0.4 mm
42
0.6 mm
43
0.4 mm
44
0.6 mm
45
0.4 mm
46
0.6 mm
47
1 mm
48
0.6 mm
49
0.4 mm
50
0.2 mm
51
